# Discrimination and Psychosocial Well-Being of Migrants in Spain: The Moderating Role of Sense of Community

**DOI:** 10.3389/fpsyg.2020.02235

**Published:** 2020-09-18

**Authors:** Alba García-Cid, Luis Gómez-Jacinto, Isabel Hombrados-Mendieta, Mario Millán-Franco, Gianluigi Moscato

**Affiliations:** ^1^Department of Social Psychology, Social Work, Social Anthropology and East Asian Studies, University of Málaga, Málaga, Spain; ^2^Faculty of Psychology, University of Málaga, Málaga, Spain; ^3^Faculty of Social and Labour Studies, University of Málaga, Málaga, Spain

**Keywords:** migrants, discrimination, psychosocial well-being, sense of community, satisfaction with life, psychological distress, social exclusion

## Abstract

The discrimination migrants perceive during their adaptation process is one of the main sources of stress and it affects their well-being, health and integration severely. The present study analyses how the sense of community (SOC) can have a protective effect against the perception of discrimination and its negative consequences by verifying the following theoretical model: discrimination predicts three indicators of psychosocial well-being (psychological distress, satisfaction with life and feelings of social exclusion). Furthermore, the theoretical model proposed also considers the hypothesis that SOC has a moderating role on the effect of perceived discrimination regarding the three variables mentioned above. 1714 migrants from Eastern Europe, Africa and Latin America who live in Málaga, Spain, participated in the study. Data were collected using random-route sampling and survey methodology. After carrying out multiple regression analyses, using the PROCESS tool in SPSS 20, the theoretical model was verified: SOC reduces the negative effects of perceived discrimination for the variables psychological distress, satisfaction with life and social exclusion feelings. Therefore, migrants who have a greater SOC experience fewer negative consequences, as compared to those with a lower SOC, for whom the consequences of such variables are more negative. These results highlight the importance for migrants to rebuild social networks in the host country and develop a good SOC. Results also allow the development of intervention patterns to favor positive interactions between native population and migrants.

## Introduction

Spain has received large numbers of migrants over the past three decades. According to the Spanish National Statistical Institute (2019), 10% of the population in Spain is foreign. Traditionally, the Spanish population reacted positively toward migrants. However, discriminatory attitudes toward migrants have appeared after the recent recession. According to a survey conducted by the Spanish Sociological Research Centre (2017), more than 50% of Spanish people believe migrants receive more than what they contribute to the Spanish society and when it comes to employ personnel, they prefer to hire Spanish people than migrants. However, this is not only the case in Spain: record levels of displaced people were reached in 2017 worldwide, with a total of 65 million (Allen et al., 2018). In fact, it is expected that by 2050 the number of displaced people will reach 230 million, who leave their countries of origin in search of improving their economy, education, and employment and personal opportunities (International Organization for Migration, 2008). Over the past decade, most common destinations for migrants have been the US, Canada, Australia and New Zealand; Asian countries such as Japan, Singapore, Hong Kong, Malaysia and the Gulf States; and European countries (Gorter et al., 2018). In such global context, there are social stratification and discrimination axes, both at individual and institutional level, which divide the migrant population into socially included and excluded, whether this is due to ethnicity, gender, or place of origin (Bassel, 2010; Dhamoon, 2011). This situation of global structured discrimination has given rise to actions that oppose racialization and express the need for change toward achieving a society with equal opportunities, security and well-being for all citizens (see Black Lives Matter, Jee-Lyn García and Sharif, 2015). So much so, that from the beginning of the 20^th^ century, many migrant families in the US decided to change their names in an attempt to shear off their ethnic trace and that of their children, which caused them to be victims of discrimination at school, work and home (Biavaschi et al., 2013). As noted by Lamont (2009), the perception of ethnic discrimination accentuates borders and hinders migrants’ social cohesion in host countries.

Discrimination can be understood as a social network system whose aim is to limit the economic, political and social opportunities of a specific collective, through subtle and clear behaviors (Bobo and Fox, 2003). This discrimination is promoted by prejudices and stereotypes that are aesthetic, economic, social, religious or cultural, amongst others (Quillian, 2006; del Olmo, 2009). In this sense, discrimination harms well-being in many aspects of life: it makes access to health care more difficult, as well as school learning, finding employment, housing, etc. More specifically, discrimination places individuals in a disadvantaged position in society (Schmitt and Branscombe, 2002), it excludes them from their groups of reference, and it causes in them feelings of helplessness and rejection (Wirth and Williams, 2009).

Discrimination and rejection perceived in the host country is one of the main sources of stress suffered by migrants and it seriously harms their well-being (Allport et al., 1954; Paradies, 2006; Schmitt et al., 2014). It consists of a continuing discriminatory abuse which produces social stress and psychological distress, which ultimately harms migrants’ mental and physical health, it deteriorates their self-esteem and it produces alienation and feelings of rejection (Sellers et al., 2003; Bhugra and Ayonrinde, 2004; Bhugra and Becker, 2005), thus reducing discriminated individuals’ quality of life (LeBel, 2008; Wang et al., 2010). This type of stress is one of the predictor variables for mental health (Jasinskaja-Lahti et al., 2006; Schunck et al., 2015; Alvarez-Galvez, 2016; Urzúa et al., 2016; González-Rábago et al., 2017) and it is related to depressive illnesses (Finch et al., 2000; Liebkind and Jasinskaja-Lahti, 2000) anguish, anxiety (Mickelson and Williams, 1999) and deterioration of self-esteem (Verkuyten, 1998; Jia et al., 2017), self-perception of health (Borrell et al., 2010; Agudelo-Suárez et al., 2011; Brondolo et al., 2011; Gil-González et al., 2014; Nakhaie and Wijesingha, 2015), satisfaction with life (Branscombe et al., 1999; Brown et al., 2000; Brown, 2001), and physical well-being (Krieger and Sidney, 1996; Finch et al., 2001; Pascoe and Smart Richman, 2009).

When individuals decide to migrate, they usually leave their families behind as well as their closest social ties and support networks that protect their health and well-being (Han and Choe, 1994; Runyan et al., 1998; García-Cid et al., 2017; Millán-Franco et al., 2019a). Migrants settling in a new country must adapt to the traditions and symbols of the new community, which contributes to reduce the sense of community (SOC) from their countries of origin and build a new SOC in the host country (Bathum and Baumann, 2007; Hombrados-Mendieta et al., 2019). Authors such as Phinney (1990) and Berry (1997) noted that migrants deal with both cultures independently and bidirectionally. Some migrants prefer to create social ties with the host community and keep the bonds with their communities of origin; this is known as acculturation strategy (Berry, 1980). However, other migrants show negative feelings toward their communities of origin and prefer to adapt and benefit from the host community; for instance, Afghan women who join resistance organizations from the host community (Brodsky, 2009). This adaptation process has significant effects on the well-being of migrant population. The degree of social interaction and integration of migrants in the host community are key elements to predict well-being and appropriate coexistence with native population (Nauck, 2001; Birman et al., 2002; Zhang et al., 2019). Migrants’ structural integration in the education and labor systems of host countries reduce borders between groups and ethnic prejudices, in such a way that minorities which are not socio-economically integrated in host countries face more discrimination and social exclusion (Alba, 2005).

Another factor to consider when analyzing the integration and adaptation of minoritarian migrant groups is the difference between countries of origin and host countries (Jonsson et al., 2012, 2018): differences in ethnic markers such as the color of the skin, religion, values and beliefs, wealth, level of education and literacy, language and gender equality, amongst other, hinder the process of social adaptation of migrants and increases the shock between both cultures (Kim, 2017). It could be said that it constitutes a bidirectional process: those migrants who are culturally closer will face less difficulties to learn new habits and cultural abilities, which will lead to higher acceptation from the majoritarian population and they will subsequently experience less discrimination (Cuddy et al., 2008; Ward, 2009; Berry and Ward, 2016).

It would be also important to highlight in this study the length of residence in the host country. This variable leads to contradictory results: length of residence has been traditionally considered to relate to higher adaptation and well-being of the collective, thanks to acculturation strategies that would reduce the stress suffered by migrants (Millán-Franco et al., 2019c). However, new research suggests that the length of residence can have a negative impact in the well-being of migrants: for instance, migrants who acquire new habits from the native population and reduce their physical activity or change their diets. Also caused by deficient access to health services or medical attention or due to adverse working conditions during their first years of settlement (Subedi and Rosenberg, 2014; Ronda-Perez et al., 2019). Bentley et al. (2019) suggest that adverse conditions such as discrimination suffered in the host country decrease the mental health and well-being of the collective, with this correlation being significant after 7 years of residence in the host country.

McMillan (1976, p. 9) defined SOC as “a feeling that the members of a community have in relation to their belonging to a community, a feeling that members worry about each other and that the group is concerned about them, and a shared faith that the needs of the members will be satisfied through their commitment of being together.” McMillan and Chavis (1986) suggested that the SOC is a multidimensional concept that consists of membership, influence, integration and fulfillment of needs, and shared emotional connection.

The SOC is absolutely crucial for migrants, who build new communities and interaction contexts during their adaptation process (Bathum and Baumann, 2007). Isolation from the community has been related to health decline, depressive symptoms and even suicidal thinking in migrants (Pickett and Wilkinson, 2008; Mair et al., 2010; Pan and Carpiano, 2013). Those migrants who feel excluded from the host community, feel discriminated and perceive rejection from the native population experience a decline in physical health and higher incidence of mental health problems, as compared to those migrants who establish stable social and emotional relations and belong to the protective environment provided by the community (Cochrane and Stopes-Roe, 1977; Harding and Balarajan, 2001; Albanesi et al., 2007; Cicognani et al., 2008; Sharapova and Goguikian, 2018).

Sense of community is also related to the degree of integration in the host country and migrants’ satisfaction with life (Cooper et al., 1992; Herrero et al., 2011; Hombrados-Mendieta et al., 2013; Millán-Franco et al., 2019b). It plays a crucial role in the well-being of the collective (Sarason, 1986; Malone, 2001). In this line of thought, many studies points to SOC as key for the migrant collective to experience the migration process positively, feeling part of the host community, benefiting from the available resources and improving their psychological well-being. SOC would also reduce high levels of psychological distress caused by the migration process (Cohen et al., 2000; Bhui et al., 2012; Bak-Klimek et al., 2015; Bobowik et al., 2015).

Research has also proved that SOC relates positively to citizen engagement (Davidson and Cotte, 1989; Perkins et al., 1990). Social cohesion is understood as an indicator of good relations in the community and between neighbors. It also relates positively to the quality of life of migrants, which promotes their participation in community activities voluntarily and it encourages informal social control (Sampson et al., 1997; Vega et al., 2011; Lee et al., 2017). Positive interactions, integration and solidarity between the endogroup and the exogroup are thus favored. They contribute not only to defend themselves from perceived threats but also as a way to reach those factors that could increase their well-being and quality of life in the host country (Ford and Beveridge, 2004; Ng et al., 2015). Social cohesion is hence considered a key factor in the adaptation process for migrants, which is tightly linked to the feeling of belonging to the host community (Amit and Bar-Lev, 2015). It is also one of the main reasons that makes migrants decide to stay in the host country and not migrate again (Kilpatrick et al., 2011; Kilpatrick et al., 2015). On the contrary, those collectives that are marginalized and excluded to a greater extent feel less attached and committed toward the host community (Wray-Lake et al., 2008). It is also necessary to mention how the cultural shock affects migrants’ citizen commitment and the cohesion between communities: ethnic diversity between groups, economic diversity, language, and religious diversity could mediate the confidence between migrants and native population, harming the contact in the neighborhood and the quality of relations between those groups that are culturally further (Lancee and Dronkers, 2011).

As it has already been mentioned, migrants are forced to face multiple changes and highly stressful situations that lead to health problems and a decline of their psychosocial well-being, such as unemployment, poor housing, instability, discrimination, isolation, network interruption and separation from their social ties or family (Grieco, 1998; Hagan, 1998). However, literature suggests that SOC works as a protective factor for the migrant population. Those who are able to rebuild their social networks and relations with the new community will not suffer so many health problems, their satisfaction with life will be higher (Farrell et al., 2004; Simich et al., 2005) and the effect from the stress related to the process of acculturation will be eased (Golding and Burnam, 1990; Jibeen and Khalid, 2010; Hombrados-Mendieta et al., 2013). Positive social cohesion and contact are key factors for migrants (Berkman and Glass, 2000; Kawachi and Berkman, 2000); integration in neighborhoods would mediate in economic instability, health problems and well-being (Browning and Cagney, 2003). As pointed out by Fisher and Sonn (2002, p. 12) “without these, the person and the group flounder.”

### The Present Study

The present study aims at verifying whether SOC acts like a shock-absorbing element against discrimination. It also aims at confirming whether it can provide a space to express identities and help cope with the changes of our worlds, as expressed by authors such as Fisher and Sonn (2002). SOC would act as a moderating variable of the negative effects from discrimination and, therefore, it would help increase migrants’ psychosocial well-being.

One of the main aims of the present study is to analyze the negative effects from discrimination based on factors that relate not only to individuals but also to communities. Most studies have paid greater attention to personal well-being and have analyzed the negative consequences caused by discrimination on variables such as self-esteem (Liu and Zhao, 2016), self-control, confrontation abilities or level of studies as protective factors against discrimination (Dion et al., 1992). However, there is a lack of studies that analyze jointly the role of personal and community factors in order to reduce the effects of discrimination.

When it comes to delving into the negative relation between discrimination and the well-being of the migrant collective, it is necessary to look closer to gender differences (Kim and Noh, 2014; García-Cid et al., 2020). Several authors find that discrimination harms the mental health and psychological distress of women to a greater extent (Turner and Avison, 2003; Flores et al., 2008; Bernard et al., 2017), even if they are exposed to fewer levels of discrimination (Hahm et al., 2010). Other authors note that women are victims of discrimination more frequently as opposed to men. This situation is aggravated in migrant women, who are victims of discrimination three times over, that is, due to ethnicity, gender and social class (Mbiyozo, 2018). For these reasons, the present study takes into consideration the differences between men and women in the analysis and interpretation of the results.

In the present study, **sense of community** (SOC) is considered the epicentre of the investigation. It advocates for the protective effect of SOC against the **perception of discrimination** and its negative consequences on the variables *psychological distress*, *satisfaction with life*, and *social exclusion feelings*.

**FIGURE 1:**
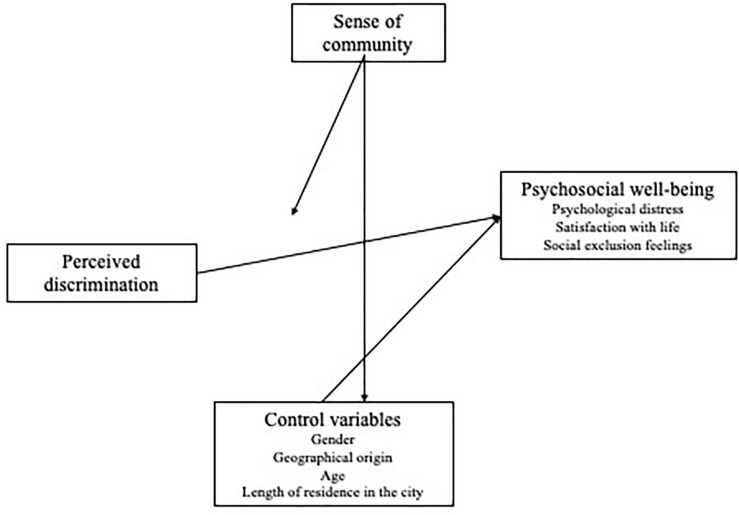
Theoretical model.

Figure 1 shows the theoretical model suggested in this study:

*Hypothesis 1*: contemplates that **perceived discrimination** predicts three indicators of psychosocial well-being: psychological distress, satisfaction with life and social exclusion feelings. Perceived discrimination is expected to relate to lower levels of satisfaction with life and higher levels of psychological distress and social exclusion feelings.

*Hypothesis 2*: **sense of community** is predicted to relate to higher levels of satisfaction with life and lower levels of psychological distress and social exclusion feelings.

Along with this direct positive effect on psychosocial well-being,

*Hypothesis 3*: suggests the moderating effect of SOC: it is expected to moderate the negative effects of discrimination. The negative effects resulting from discrimination on the three indicators of psychosocial well-being would be lower if migrants have higher SOC and they would be higher if migrants’ SOC is lower.

In order to clearly test such moderating effect of SOC,

*Hypothesis 4*: propose that demographic factors could have an impact on this effect. Gender, geographical origin, age and length of residence in the city are included in the model as covariables.

## Materials and Methods

### Participants

The participants consisted of 1714 migrants from Eastern Europe (31.6%), Africa (33.2%), and Latin America (35.2%). In total, there were 48.7% men and 51.3% women. The average age was 33 years old (*SD* = 12.31; range = 16–74). They had lived in the city of Málaga for an average of 10.35 years (*SD* = 7.30; range, from less than 1 to 53 years). 34.3% were married, 47.8% were single, 8.5% had an unmarried partner, 7.4% were separated or divorced and 2% were widowed. 33% had attended or completed primary school; 38% secondary school; 18% had attended university but not completed a degree and 11% had a university degree. 56.4% were employed and 43.6% were unemployed. This distribution is representative of the distribution of migrants in the city in which this study was conducted, as referenced by the 2017 census data.

### Procedure

Data were collected using a random-route sampling and survey methodology. Boundaries were established for each of the neighborhoods selected and random route sampling was used to designate the blocks, streets, sidewalks, and so on, in each neighborhood. Carefully trained interviewers administered the surveys. These surveys were collected in the city of Málaga, Spain, within 11 city districts. This involved sampling from the 11 municipalities of Málaga with the greatest concentration of migrants. Questionnaires applied to non-Spanish-speaking people were translated into their language of origin by native speaker**s** (“the translator”) who had a full command of Spanish. In order to ensure both languages matched, the translator read the questions and ensured the objective of each sections had been understood. Answers were subsequently registered by the interviewer according to the methodology suggested by the transcultural research by Páez and Vergara (2000). The surveys were conducted at immigrant associations, businesses, meeting places, and Social Service Centres located within each district. All participating migrants were volunteers and signed an informed consent. No incentives were offered for their participation. The Ethical Commission of the University of Málaga (CEUMA: 37−2016−H) determined the suitability of the protocol.

### Measures

#### Demographic Form

For the participating migrants, data were collected on: country of origin, age, gender, and time of residence. Descriptive statistic can be seen in Table 1.

**TABLE 1:** Correlations, descriptive statistics and Cronbach’s alpha for the study variables.

	1	2	3	4	5	6	7	*M*	*SD*	Range	Skewness	α
(1) Perceived discrimination	…							1.43	0.49	1–4	0.833	0.85
(2) Sense of community	−0.29**	…						3.19	0.92	1–5	–0.127	0.89
(3) Psychological distress	0.33**	−0.27**	…					11.15	5.54	0–34	0.858	0.84
(4) Satisfaction with life	−0.36**	0.44**	−0.45**	…				4.34	1.38	1–7	–0.182	0.90
(5) Social exclusion feelings	0.43**	−0.28**	0.28**	−0.36**	…			2.53	0.93	1–5	0.328	0.70
(6) Age	0.01	0.10**	0.02	−0.05*	0.05*	…		33.88	12.31	16–74	0.573	
(7) Length of residence in the city	−0.175**	0.18**	−0.12**	0.17**	−0.16**	0.41**	…	10.35	7.30	0–53	–0.970	

*p < 0.05; **p < 0.01.

#### Perceived Discrimination

The questionnaire on discrimination is based on Krieger’s design (Krieger et al., 2005). Experiences of discrimination: validity and reliability of a self-report measure for population health research on racism and health. This scale is backed by more than 500 studies and it was intentionally used to measure perceived discrimination, whether caused by ethnicity, gender or age, both individually and combined (Llácer et al., 2009; Krieger, 2014). Participants were asked: *During the last year, have you felt discriminated in any of the following situations?* Nine situations that can occur at the work environment, when accessing public services, education or health, when treated by police officers, when accessing housing, at shops, in the street, etc. were presented. Responses were recorded using the Likert scale: never (1), sometimes (2), often (3), many times (4). The Confirmatory factor analysis (CFA) by generalized least squares (GLS) of the items showed that one factor explained 45% of the variance. All items exceeded coefficients over 0.60, except for those regarding discrimination within the family, which showed a coefficient of 0.47. The model had an appropriate goodness of fit, χ^2^ = 680.33, d.f. = 27, *p* < 0.0001. Internal consistency was also good (see Table 1).

#### Sense of Community

The Brief Sense of Community Scale created by Peterson et al. (2008). This instrument is based on the components of the SOC model provided by McMillan and Chavis (1986): fulfillment of needs, group membership, influence and emotional connection. This scale has been widely used in social science, both in Spain (i.e., by Hombrados-Mendieta et al., 2013) and internationally (i.e., Au et al., 2020). The questionnaire consists of eight items that are measured through a Likert-type scale: (1 = Strongly disagree; 5 = fully agree). The SOC Global Index was calculated by summing the eight items. The CFA of items showed that one factor explained 58.13% of the variance. All factor loadings are higher than 0.60, except for one item, which showed 0.47; goodness of fit is high (χ^2^ = 545.26, d.f. = 20, *p* < 0.0001). As it can be seen on Table 1, the questionnaire has a good internal consistency.

#### Psychological Distress

The Spanish version of the Goldberg General Health Questionnaire (GHQ-12) was used (González et al., 2013). This instrument focuses on the interruptions of normal psychological functioning, rather than psychopathological traits. It includes within its scope personality disorders or patterns of adjustment related to distress. This questionnaire has proved to be an effective tool for the evaluation of mental health symptoms in clinical patients and the general population (González-Castro and Ubillos, 2011; Martos-Méndez et al., 2020). It consists of 12 items which are answered through a four-point Likert-type scale (0–3) ranging from (0) = Not at all, to (3) = Much more than usual (e.g., “*Have you felt constantly overwhelmed and stressed?*”). The CFA with GLS estimation shows that one factor explained 37.35% of the variance; factor loadings range from 0.33 to 0.73, with the majority exceeding 0.50. Goodness of fit is appropriate (χ^2^ = 533.58, d.f. = 54, *p* < 0.0001) and Cronbach’s α is good (Table 1).

#### Satisfaction With Life

The five-item Satisfaction with Life Scale (SWLS), developed by Pavot and Diener (1993b), was used to assess life satisfaction or the cognitive component of well-being. This scale includes general items for interviewed participants to assess the domains of their lives according to their own values, thus reaching a global assessment of their own life satisfaction. This scale has been widely used because its normative data include highly diverse populations such as convicts, older adults, abused women or intercultural collectives, amongst other (Pavot and Diener, 1993a; Elgorriaga et al., 2016). The confirmatory factor analysis of this questionnaire shows that only one factor explains 71.6% of the variance. All items have loadings over 0.70 and the scale has an appropriate goodness of fit index (χ^2^ = 68.96, d.f. = 5, *p* < 0.0001). The scale has a Cronbach’s α that is also good (Table 1).

#### Social Exclusion Feelings (Moscato et al., 2014)

This scale consists of four items using a five-point Likert-type scoring system (1 = strong disagreement; 5 = strong agreement). It assesses the experience of feeling excluded in the host country. This scale has been used with migrant population in Spain and it is an appropriate instrument to assess rejection feelings experienced in host communities (“*I receive few services due to being a foreigner in Spain”; “I sometimes feel excluded or ignored in Spain”; “I sometimes feel like I am treated with little respect”; “It is difficult to find work with my level of education in Spain*”). The scale has a Cronbach’s α = 0.70. Only one factor explained 54.8% of the variance; two items have coefficients over 0.80 and two over 0.30. The scale has a moderated Cronbach’s alpha (Table 1).

### Plan of Analysis

Analyses were carried out using IBM SPSS Statistics 20. Descriptive statistics were initially calculated, along with correlations between variables and differences based on gender and geographical origin.

In order to calculate the network of relations from the suggested model in Figure 1, multiple regressions were carried out with perceived discrimination, SOC and interaction both as predictor variables; gender, geographical origin, age and time of residence in the city were considered covariables; the rest of variables were considered as dependent from the regression equation. These analyses were conducted using the PROCESS version 3.4.1 tool in SPSS 20 (Hayes, 2018). Before carrying out regression analyses, a log transformation was performed, Ln (x), on perceived discrimination and time of residence, with the aim of improving their skewness indexes, with values of 1.53 and 1.27 respectively, which are notably higher than 0. Their skewness was improved after the log transformation, as it can be seen on Table 1. The remaining variables are also shown in it; all remained below ∓ and close to 0. After the transformations all variables were standardized. Dummy variables were created for gender (0 = woman; 1 = man) and geographical origin. Two dummy variables were created for geographical origin: migrants from Eastern Europe are codified as 1 in the first dummy variable and the remaining as 0; Latin American migrants are codified as 1 in the second variable and the remaining as 0. Therefore, African migrants are considered as the reference category, which is 0 in both dummy variables.

Calculations were exclusively carried out with those participants who completed all scales and without substituting lost values. *N* = 1590 for the regression of psychological distress and *N* = 1636 for the regressions of life satisfaction and exclusion feelings. Multicollinearity diagnostics indicated that the variance inflation factor (VIF) did not exceed in none of the cases 1.1; this indicates an acceptable level of multicollinearity between variables.

## Results

### Descriptive Analyses

Table 1 summarizes descriptive statistics, Cronbach’s alpha and correlations between the study’s variables, which were calculated without replacement. Migrants who participated in this study perceive low levels of discrimination; they state medium levels of SOC; their psychological distress and satisfaction with life is medium; their social exclusion feelings are medium.

Perceived discrimination relates negatively to SOC and satisfaction with life. It relates positively to psychological distress and social exclusion feelings. SOC relates positively to satisfaction with life. Conversely, it relates negatively to psychological distress and social exclusion feelings.

Table 2 shows the differences between variables based on geographical origin and gender. Some differences can be observed between the three groups of migrants regarding discrimination, satisfaction with life and social exclusion feelings. Migrants from Africa are the ones who experience more discrimination, as compared to migrants from Latin America, who experience the least. Migrants from Africa feel more excluded than migrants from Eastern Europe and Latin America.

**TABLE 2:** Means and standard deviations of key variables by geographical origin and gender.

	Geographical origin							Gender				
		
	Africa		Europe		Latin America			Women		Men		
								
	*M*	*SD*	*M*	*SD*	*M*	*SD*	*F*	*M*	*SD*	*M*	*SD*	*F*
Discrimination	1.55	0.52	1.40	0.49	1.35	0.43	25.20**	1.41	0.48	1.46	0.51	5.36*
Sense of community	3.19	0.95	3.20	0.92	3.19	0.91	0.04	3.27	0.94	3.11	0.91	13.60**
Psychological distress	11.28	5.38	11.30	5.60	10.91	5.65	0.90	11.16	5.62	11.14	5.50	0.01
Satisfaction with life	4.21	1.37	4.28	1.42	4.54	1.34	9.41**	4.48	1.37	4.20	1.38	17.43**
Exclusion feelings	2.73	0.93	2.49	0.97	2.40	0.87	18.99**	2.52	0.94	2.55	0.94	0.35

*p < 0.05; **p < 0.01.

Regarding gender, there are differences in satisfaction with life but not in psychological distress and social exclusion. Men perceive more discrimination than women. The latter have higher SOC than the former. Women’s satisfaction with life is higher than men’s.

### Predicting Psychosocial Well-Being Variables

Results from moderating regression analyses are shown in Table 3. Perceived discrimination is considered predicting variable, SOC as moderator and those related to psychosocial well-being are considered dependent variables. For all cases, gender, geographical origin, age and length of residence in the city are considered as covariables. As it can be observed, regressions from each dependent variable are statistically significant.

**TABLE 3:** Regressions analyses testing the moderating effects of sense of community in the relationship of perceived discrimination to psychosocial well-being variables.

	Psychological distress						Satisfaction with life						Exclusion feelings					
			
					95% CI						95% CI						95% CI	
															
	*Coefficient*	*SE*	*t*	*p*	*LL*	*UL*	*Coefficient*	*SE*	*t*	*p*	*LL*	*UL*	*Coefficient*	*SE*	*t*	*p*	*LL*	*UL*
Constant	–0.030	0.048	–0.624	0.533	–0.125	0.065	0.025	0.043	0.588	0.557	–0.060	0.111	0.116	0.045	2.601	0.009	0.029	0.204
Discrimination (DIS)	0.275	0.025	10.788	0.000	0.225	0.325	–0.244	0.023	–10.755	0.000	–0.288	–0.199	0.375	0.023	16.046	0.000	0.329	0.421
Sense of community (SOC)	–0.177	0.025	–7.104	0.000	–0.225	–0.128	0.356	0.022	15.944	0.000	0.313	0.400	–0.147	0.023	–6.389	0.000	–0.192	–0.102
DIS x SOC	–0.080	0.023	–3.498	0.000	–0.125	–0.035	0.042	0.021	2.027	0.043	0.001	0.082	–0.055	0.021	–2.597	0.009	–0.096	–0.013
Geographical origin_Dummy 1_	0.084	0.058	1.430	0.153	–0.031	0.198	–0.035	0.052	–0.675	0.500	–0.138	0.067	–0.154	0.054	–2.850	0.004	–0.260	–0.048
Geographical origin_Dummy 2_	0.039	0.057	0.684	0.494	–0.072	0.150	0.140	0.051	2.739	0.006	0.040	0.240	–0.189	0.052	–3.601	0.000	–0.292	–0.086
Gender_Dummy 1_	–0.068	0.046	–1.454	0.146	–0.159	0.024	–0.110	0.042	–2.639	0.008	–0.192	–0.028	–0.053	0.043	–1.232	0.218	–0.137	0.031
Age	0.040	0.025	1.620	0.106	–0.008	0.089	–0.113	0.022	–5.066	0.000	–0.156	–0.069	0.100	0.023	4.386	0.000	0.055	0.145
Length of residence in the city	–0.053	0.025	–2.105	0.035	–0.103	–0.004	0.068	0.023	2.994	0.003	0.023	0.112	–0.095	0.023	–4.069	0.000	–0.141	–0.049
	*R^2^* = 0.161 *F* = 38.02 *p* = 0.000 Δ*R*^2^_interaction_ = 0.006 *F* = 12.23 *p* = 0.000						*R^2^* = 0.290 *F* = 83.13 *p* = 0.000 Δ*R*^2^_interaction_ = 0.002 *F* = 4.11 *p* = 0.043						*R^2^* = 0.254 *F* = 69.42 *p* = 0.000 Δ*R*^2^_interaction_ = 0.003 *F* = 6.74 *p* = 0.009					

**FIGURE 2:**
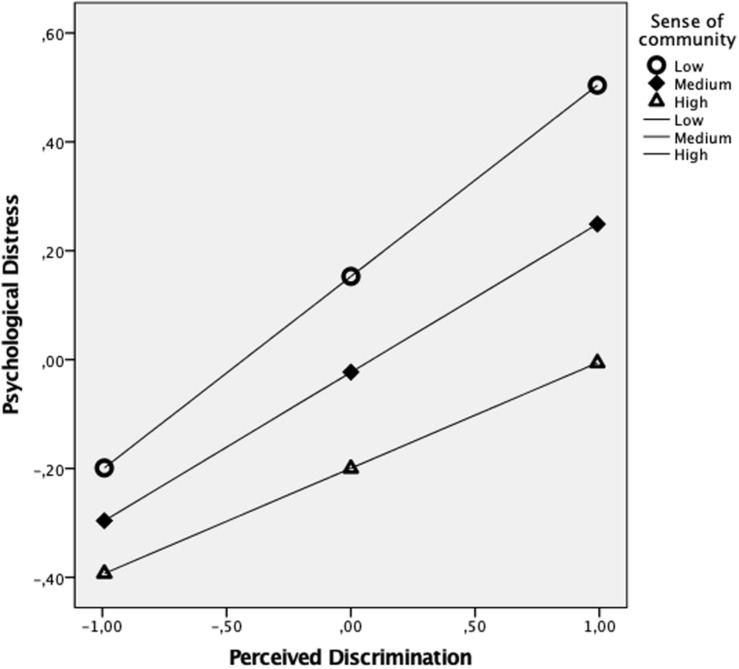
Conditional effect of perceived discrimination on psychological distress at values of sense of community.

Covariables of gender and age do not have a significant effect on **psychological distress**. SOC decreases this variable whereas discrimination increases it. The effect from the interaction of both variables is considerable. Discrimination increases psychological distress, but this decrease when SOC is higher (see Figure 2).

All covariables have significant effects on **satisfaction with life:** migrants from Latin America, women, younger migrants and those who have lived longer in the city feel more satisfied with life. This variable is positively influenced by SOC and negatively by discrimination; interaction between both variables is considerable. The negative effects of discrimination on satisfaction with life decrease when migrants have high SOC (see Table 4).

**TABLE 4:** Conditional effect of Perceived discrimination on psychosocial well-being variables at values of the Sense of community.

	Psychological distress						Satisfaction with life						Exclusion feelings					
			
					95% CI						95% CI						95% CI	
															
*Sense of*																		
*community*	*Effect*	*SE*	*t*	*p*	*LL*	*UL*	*Effect*	*SE*	*t*	*p*	*LL*	*UL*	*Effect*	*SE*	*t*	*p*	*LL*	*UL*
Low	0.354	0.032	11.013	0.000	0.291	0.417	−0.285	0.029	−9.941	0.000	−0.342	−0.229	0.430	0.030	14.519	0.000	0.372	0.488
Medium	0.275	0.025	10.788	0.000	0.225	0.325	−0.244	0.023	−10.755	0.000	−0.288	−0.199	0.375	0.023	16.046	0.000	0.329	0.421
High	0.195	0.036	5.405	0.000	0.124	0.266	−0.202	0.032	−6.273	0.000	−0.266	−0.139	0.320	0.033	9.634	0.000	0.255	0.385

Finally, for the case of **social exclusion feelings**, all covariables except for gender have significant effects. Migrants from Eastern Europe and Latin America feel less excluded, as compared to those who come from Africa. Older migrants feel more excluded, as compared to those who have lived longer in the city, who feel less excluded. Discrimination as independent variable increases exclusion feelings significantly, whereas SOC decreases them. Interaction between the independent variable and the moderating variable is considerable; social exclusion feelings caused by perceived discrimination decrease when migrants have higher levels of SOC (see Table 4).

## Discussion

The present study suggests a theoretical model in which **perceived discrimination** predicts three indicators of psychosocial well-being (*psychological distress*, *satisfaction with life* and *social exclusion feelings*) in the migrant population. Along with this direct relation of discrimination, the model also includes the moderating effect of **sense of community**. The SOC plays a moderating role on the effect of perceived discrimination and it suggests that the negative effects of discrimination on the indicators of psychosocial well-being are lower when migrants have a higher SOC. Conversely, such negative effects are higher when migrants’ SOC if lower.

After testing this theoretical model, results achieved show the following:

*Hypothesis 1*: As it was expected, **perceived discrimination** relates to lower levels of *satisfaction with life*, and higher levels of *psychological distress*. These results match with results from studies that confirm perceiving discrimination is the main trigger factor for stress amongst migrants, leading to a decline in mental health (Finch et al., 2000; Liebkind and Jasinskaja-Lahti, 2000; Jasinskaja-Lahti et al., 2006; Schunck et al., 2015; Alvarez-Galvez, 2016; Urzúa et al., 2016; González-Rábago et al., 2017), satisfaction with life (Branscombe et al., 1999; Brown et al., 2000; Brown, 2001) and physical well-being (Krieger and Sidney, 1996; Finch et al., 2001; Nazroo, 2003; Pascoe and Smart Richman, 2009). Perceived discrimination is also proved to predict higher *social exclusion feelings* (Herz and Johansson, 2012; Oxman-Martinez et al., 2012).

*Hypothesis 2*: Results obtained from direct analyses prove that **SOC** relates to higher levels of *satisfaction with life* and less *psychological distress*. Therefore, isolation from the community is linked to a decline in health and the appearance of mental health problems (Pickett and Wilkinson, 2008; Mair et al., 2010; Pan and Carpiano, 2013). Developing a good SOC relates positively to integration (Cooper et al., 1992; Herrero et al., 2011; Hombrados-Mendieta et al., 2013) and a good satisfaction with life (Sarason, 1986; Malone, 2001). As it was expected, SOC is also linked to lower levels of *social exclusion feelings*. SOC is considered crucial during the process of integration in the new country (Nauck, 2001; Birman et al., 2002).

After analyzing direct effects,

*Hypothesis 3* suggested to delve into the study’s variables by analyzing the moderating effects of SOC: significant interactions were found between psychological distress and satisfaction with life. Although **discrimination** increases *psychological distress* and decreases the migrants’ *satisfaction with life*, the **SOC** acts like a shock-absorbing factor, easing these negative effects and preserving individuals’ well-being. As it has been suggested by previous studies, the SOC is a strong protective factor against mental health problems and satisfaction with life. It takes in individuals under the protective environment of the community and therefore stable social relations between migrants and native population should be promoted (Cochrane and Stopes-Roe, 1977; Cooper et al., 1992; Farrell et al., 2004; Simich et al., 2005; Albanesi et al., 2007; Basabe et al., 2009; Herrero et al., 2011; Hombrados-Mendieta et al., 2013; Liu and Zhao, 2016). These relations contribute to reduce the appearance of depressive symptoms and suicidal thinking caused by isolation from the community and perceived discrimination (Clare, 1974). *Social exclusion feelings* caused by perceived discrimination decrease when migrants have higher SOC. This means that interaction between the independent variable and the moderating variable is significant. It could be said that in our study, SOC acts as a buffering variable and protective factor against the negative effects caused by discrimination and rejection experienced in the host country, which would positively affect migrants’ integration and adaptation. These results match with studies that confirmed migrants were able to rebuild their social networks in the host country and that SOC acted as a protective factor against the negative effects of discrimination and stress caused by the acculturation process (Golding and Burnam, 1990; Jibeen and Khalid, 2010; Hombrados-Mendieta et al., 2013). Results also match other studies that point to the importance of social cohesion and positive contact with native population and neighbors (Berkman and Glass, 2000; Kawachi and Berkman, 2000; Fisher and Sonn, 2002; Browning and Cagney, 2003). This might be due to the collective’s needs being satisfied by the native community to a larger extent than by their fellow citizens. Native population are sources of information about the traditions and habits of the host country (Domínguez-Fuentes and Hombrados-Mendieta, 2012; García-Cid et al., 2017). Therefore, when SOC develops thanks to positive interactions with the native population it plays a vital role in the process of integration and well-being faced by migrants (Searle and Ward, 1990).

*Hypothesis 4*: based on the results obtained from analyzing covariables, older migrants feel more excluded, but the length of time spent reduces such feelings. According to what other studies suggest, the longer the time an individual spends in the host society, the more opportunities he or she has to create ties with the community and feel part of it (Prezza et al., 2008; Millán-Franco et al., 2019a). Migrants from Eastern Europe and Latin America feel less excluded as compared to African migrants, who express feeling more discrimination. This is in line with other studies which suggest that the cultural shock is decisive for the adaptation process (Jonsson et al., 2012; Kim, 2017; Jonsson et al., 2018); similar results were found in other studies carried out in Málaga (Cofrades, 2010) and Spain (Ioé, 2003), where African migrants showed worst indexes of well-being, health, social support and life satisfaction, amongst other, as well as feeling more discriminated. When cultural values, symbols and, sometimes, even language are shared, there is less probability to suffer discrimination from the native population (Nesdale and Mak, 2003; Jasinskaja-Lahti et al., 2006; Cuddy et al., 2008; Ward, 2009; Berry and Ward, 2016). Regarding gender differences, men are observed to perceive more discrimination, while women express higher SOC and SWL. These results are in line with those studies which noted the positive relation between SOC and SWL in migrants (Farrell et al., 2004; Simich et al., 2005; Herrero et al., 2011; Hombrados-Mendieta et al., 2013; Millán-Franco et al., 2019b). Furthermore, they show how discrimination deteriorates SWL and hinders social cohesion and feeling part of the host community, which is less in men (Wray-Lake et al., 2008; Amit and Bar-Lev, 2015). Another possible explanation for the results obtained is that, as pointed out by previous studies, men perceive more discrimination than women, while women deal better with unjust treatment and internalize their symptoms and their status is lower in society (Karlsen and Nazroo, 2002; Himle et al., 2009), being victims of the mentioned three fold discrimination (Szymanski and Lewis, 2015; Neblett et al., 2016).

## Conclusion

The theoretical model suggested has been verified: the SOC plays a moderating role on the negative effects of perceived discrimination regarding the variables of psychological distress, satisfaction with life and social exclusion feelings. The effects of these variables are lower when migrants have higher SOC, whilst consequences are more negative for migrants who have low SOC.

Results obtained from the study highlight how important for the migrant population it is to rebuild social networks in the host country and develop a good SOC. Therefore, designing interventions to strengthen positive interactions between the native and the migrant population is suggested.

Before concluding, some limitations of the study should be noted. Data were collected using self-report questionnaires. When self-report questionnaires are applied, the researcher assumes that participants’ responses accurately reflect their feelings (Heppner et al., 1992). In order to compensate this limitation, the aim is to address migrants through individual interviews or discussion groups in future studies. These qualitative techniques would complement and improve the empirical study carried out. However, these techniques add some complexity to the analysis because numerous elements make contacting the different sample groups and the possibility to carry out meetings in different moments more difficult. For instance, the collective’s own idiosyncrasy, the fact that migrants move throughout the country in search of better life and work conditions, the role played by women in some cultures or because due to their irregular situation, they prefer to stand aside (Maya, 2001).

In addition, these results may not adequately reflect the association between these variables in other countries and even regarding the different regions of Spain, and thus it would be of interest to replicate these results in countries other than Spain and other Spanish cities with different social and economic features. Therefore, a more complex research is suggested for future studies, which would broaden the areas of study. For instance, throughout the Andalusian territory or other relevant areas in terms of migrant population, such as Madrid or Catalonia.

It should also be noted that the present study used a cross-sectional design, and thus caution should be exercised when making causal inferences based on the data available. Moreover, as Holmbeck (1997) remarks, the relations between the independent variable, mediator, and outcome may not necessarily be causal. However, the present study provides useful data that help deepen our understanding of the relations between these variables.

## Data Availability Statement

The raw data supporting the conclusions of this article will be made available by the authors, without undue reservation.

## Ethics Statement

The studies involving human participants were reviewed and approved. In conducting the study, accepted principles of ethical and professional conduct have been followed (Reference number: CEUMA: 37-2016-H). We obtained ethical approval for the research from the Ethics Committee of the University of Málaga. All subjects gave written informed consent in accordance with the Declaration of Helsinki. The protocol was approved by Ethics Committee of the University of Málaga.

## Author Contributions

AG-C, IH-M, and LG-J contributed to conception and design of the work, collected and analyzed the data, performed substantial contributions to revising the work critically, and wrote the manuscript. MM-F and GM analyzed the data and wrote the manuscript. All the authors involved approved the final version of the manuscript to be published.

## Conflict of Interest

The authors declare that the research was conducted in the absence of any commercial or financial relationships that could be construed as a potential conflict of interest.
